# Comparison of Phenolic Compound Separations by HPTLC and PPEC with SDS as the Mobile Phase Component

**DOI:** 10.1155/2019/6845340

**Published:** 2019-01-10

**Authors:** Beata Polak, Adam Traczuk, Marta Kamińska, Małgorzata Kozyra

**Affiliations:** ^1^Department of Physical Chemistry, Medical University of Lublin, Chodźki 4A, 20-093 Lublin, Poland; ^2^Chair and Department of Pharmacognosy with Medicinal Plant Unit, Medical University of Lublin, Chodźki 1, 20-093 Lublin, Poland

## Abstract

The application of the surfactant (sodium dodecyl sulphate, SDS) as the component of the water-organic mobile phase in thin-layer chromatography and pressurized planar electrochromatography is presented. The influence of various variables on the separation of various phenolic compounds (flavonoids and phenolic acids) as model compounds with systems containing surfactant is discussed. The effect of concentration of butanol and SDS as well as pH of the mobile phase buffer on migration distance of the solute zones is investigated. The presence of SDS in the eluent affects the butanol solubility in the mobile phase. It allows using higher organic solvent concentration systems compared with the mode without surfactant. The amount of SDS in the eluent has the effect on the solute retention, whereas the eluent buffer pH affects the migration distances of ionisable phenolic acids both in HPTLC and PPEC. The migration distances of flavonoid glycosides are considerably longer than those of pure flavonoids. Considering second group of investigated solutes, derivatives of the benzoic acid migrate longer distances in comparison with the cinnamic acid ones. In addition, in the majority of experiments, ionisable compounds (phenolic acids) migrate longer distances in PPEC than nonionisable compounds (flavonoids). Additionally, the order of solutes differs in the PPEC and HPTLC system.

## 1. Introduction

Surfactants (surface active agents, amphiphiles) are molecules bearing both polar (hydrophilic) and nonpolar (long-chain, hydrophobic) regions in their molecules. Due to this phenomenon, they possess dual character and are able to reduce the interfacial tension and undergo self-association. The latter behaviour causes formation of aggregates (micelles) when the critical micelle concentration is exceeded. Amphiphiles enable solubilisation of water-insoluble compounds, increase the miscibility of different solvents, and stabilize emulsion.

The mobile phase with surfactant is involved in separation of mixtures with the use of chromatographic and electromigrational techniques. The ion-pairing chromatography (IPC) and micellar chromatography (MLC) belong to the previous mode. Micellar electrokinetic chromatography (MEKC) represents the latter.

The concentration of the surfactant in the mobile phase affects the property of eluent and the type of separation process. When the amphiphile content in the eluent is low (below the critical micellar concentration), it forms dispersed monomers and interacts with the compounds to be separated via ion-ion interactions. The type of chromatography applying such behaviour is known as ion-pair (ion-paring) chromatography. If the content of surfactant in the eluent is equal to critical micelle concentration or higher, various aggregates (micelles) of amphiphiles are formed. Micellar liquid chromatography or micellar electrokinetic chromatography employs such kinds of the mobile phase.

The various mechanisms involving in solute retention in ion-pairing chromatography have been discussed very broadly by Cecchi in her review [1]. She also has presented factors influencing the solute retention [1]. The concentrations of the ion-pairing reagent, organic solvent, and hydrogen ions (pH) in the mobile phase have an impact on the solute retention in IPC.

Various groups of organic and inorganic compounds have been separated with the use of ion-pairing liquid chromatography. The majority of applications involve high-performance liquid chromatography (HPLC) [2–5]. However, the papers on separation of solutes with the use of IPC thin-layer chromatography (HPTLC) [6–9] or IPC capillary electrophoresis (CE) [10, 11] are also available. The effect of the ion-pairing agent additive to the mobile phase on the electroosmotic flow has also been investigated in pressurized planar electrochromatography (PPEC) [12].

Higher content of amphiphiles in the mobile phase results in the formation of micelles and thus new mode of solute separation. In addition, it is possible to distinguish the kind of the separation mode (IPC or MLC) by inspecting the solute behaviour in system containing surfactant. The MLC mode has been performed when increasing the amphiphile concentration in the eluent deteriorate solute retention. The rise of the solute retention is observed at IPC mode of separation. The comprehensive review on the retention mechanisms in micellar chromatography has been presented by Ruiz-Angel and co-workers [13]. Presence of micelles in water-organic mobile phase reduces a content of organic solvent so MLC systems are suitable for green chemistry [14]. In addition, micellar eluent enhances solubility of various classes of compounds in water (e.g., alkanes, higher alcohols, or drugs). Thus, it enables using higher alcohols (usually immiscible with water) as the mobile phase component in MLC. The broad review on the effect of such solvents on the micelle formation has been presented by Zana [15]. MLC is used for separation of many solutes. The review on the application of MLC into the determination of biologically active compounds has been recently published [16]. Micellar systems are also used in microemulsion liquid chromatography (MELC). The effect of operating parameters on the separation process in MELC has been presented by Altria and coworkers [17].

Phenolic compounds are secondary plant metabolites. They possess various structures from simple ring (like phenolic acids) to more complicated system of phenol subunits, e.g., flavonoids. These compounds are ubiquitous in various plant parts. They also play a variety of roles in vegetable during growing, reproduction, protection, and other. Their protective character affects the human health [18–20] and has been applied by people against oxidative-damage illnesses such as coronary heart disease, stroke, and cancers. Normal phase chromatography is typically involved in investigation of phenolic compounds of plant origin. Nevertheless, the micellar systems can also be applied for this purpose. The comparison of the separation of several natural phenolic compounds with micellar, submicellar, and water-organic HPLC systems equipped with C-18 column has been presented by Cao and co-authors [21]. The process of the optimisation of solute separation in silico based on the experiments performed with MLC-HPLC modes has been proposed by Hadjmohammadi and co-authors in series of papers [22–24]. Combination of chromatographic response function, i.e., concentration of SDS and volume of organic modifier and acetic acid in the mobile phase, has been applied to simultaneous optimization of retention of four phenolic acids [22], five flavonoids [23], and mixture of phenolic acids and flavonoids [24].

The hyphen HPLC and MECK techniques (HPLC in the first dimension and MECK in the second dimension) have been employed for separation of mixture of 24 flavonoids and phenolic acids by Cesla and co-authors [25]. The latter technique (MECK) with the mobile phase containing various surfactants and ionic liquids has been used for separation of eight isoflavones and their determination in Radix Puerariae samples [26].

The micellar thin-layer chromatography has been applied for separation of flavonoids only by Sumina and coworkers [27].

Pressurized planar electrochromatography (PPEC) is the relatively new branch of electromigrational techniques. The solute separation mechanism in PPEC is based on two effects: partition (from chromatography) and electrophoresis. Contrary to planar chromatography in which the mobile phase movement is the result of capillary action, in PPEC the electric field action affects the eluent movement (electroosmotic flow, EOF) [28–38]. Besides the electric field magnitude, also other variables prompt the solute migration in PPEC. Deteriorate of solute retention is the result of the mobile phase composition (buffer pH, its type and concentration, kind and amount of organic solvent, and chiral or ion-pairing additives) [12, 31–36], type of the stationary phase applied [32, 34, 37], and temperature in which the experiment is carried out [38]. One of the advantages of PPEC system is prewetting of an adsorbent layer with the mobile phase solution before separation process, and then the same eluent is used to feed adsorbent layer during PPEC experiment. In this way, adsorbent-mobile phase system is practically equilibrated during separation process what makes PPEC system analogous to that of HPLC or CEC in this regard. The PPEC technique characterises high separation efficiency, short experiment time, and various selectivities in comparison with HPTLC [28–38].

In our work, we present comparison of the separation of two group test compounds (flavonoids and phenolic acids) with the use of pressurized planar electrochromatography and thin-layer chromatography with sodium dodecyl sulphate, SDS, as the component of the mobile phase. Suggested compounds possess different properties. Flavonoids are not ionisable, whereas phenolic acids are ionisable (undergo ionisation process). Thus, comparison of two techniques with different mechanisms of separation emphasises their benefits and limits. In addition, flavonoids and phenolic acids occur in plant together. Therefore, it is possible to verify the separation of genuine samples performed with each technique. Moreover, till now, the PPEC mode of separation of compounds of plant origin with the SDS as the component of the mobile phase has not been undertaken.

## 2. Experimental

### 2.1. Materials

Mobile phase solutions were prepared by mixing butanol and acetonitrile (Avantor Performance Materials, S.A. Gliwice, Poland) with buffer solution containing surfactant (sodium dodecyl sulphate, SDS, Sigma-Aldrich; St. Louis, Mo, USA). Buffers were prepared by mixing citric acid (0.1 M solution) with sodium hydrogen phosphate (0.2 M solution). The pH values of the buffer solutions have been determined with a CP-551 pH meter (Elmetron, Zabrze, Poland). All mobile phase components used in investigations were pure grade. The solvents (methanol and diethyl ether) and sodium bicarbonate used during extraction process were also purchased from Avantor Performance Materials SA (Gliwice, Poland).

HPTLC RP-18W plates of size 10 cm × 10 cm (Merck, Darmstadt, Germany) were used in the experiments. Standards of flavonoids and phenolic acids were received from Sigma-Aldrich (St. Louis, MO, USA).

The standards of phenolic acids and flavonoids were obtained from Sigma Aldrich (St. Louis, MO, USA). They were dissolved in acetone to form 0.1% w/v solution. List of investigated solutes, their p*K*_A_, and lipophilicity values are presented in Table 1.

Silicone sealant components Sarsil W, Sarsil H50, and hardener were obtained from Zakłady Chemiczne “Silikony Polskie” Sp. z O.O., Nowa Sarzyna, Poland.

### 2.2. Plant Material

Inflorescences were collected in July in the Medicinal Plant Garden of the Department of Pharmacognosy (Lublin, Poland). Plant material (*Carduus carlinoides*, *Carduus nigresens*, *Cirsium canum*, and *Ciriumde causatum*) was authenticated by a specialist on plant taxonomy, Prof. K. Głowniak (Medical University of Lublin), and deposited in the herbarium of the Department of Pharmacognosy. The herbs were dried at a temperature of 45°C and powdered. The procedure of preparation follows the conditions of the Polish Pharmacopoeia, 6th edition.

Voucher specimen is deposited in the herbarium of the Department of Pharmacognosy, Medical University of Lublin.

### 2.3. Equipment for PPEC

PPEC experiments were performed with the device, which was composed of PPEC chamber, high-voltage power supply (E 752, max. voltage 5 kV, Consort, Belgium) with ammeter and hydraulic press (Współpraca, Lublin, Poland). The PPEC procedure was performed according to that previously described [30]. The wetting time of the adsorbent layer was 20 s. Teflon foil was pressed to adsorbent layer of the chromatographic plate with pressure 32 bars.

### 2.4. Experiments

#### 2.4.1. Chromatographic Plate Preparation for PPEC Experiment

The plates were washed up with methanol by dipping in this solvent for 1 min. The plates were dried in the air and next in the oven at 105–110°C for 15 min and left in desiccator for cooling. Margin of 4 mm width of silicone sealant was formed on whole periphery of the chromatographic plates. Production of the margin on the plates was performed according to the procedure described in a previously published paper [39].

#### 2.4.2. Application of Investigated Compounds

Application of solutes on the chromatographic plates was performed using the aerosol applicator ATS 4 (Camag, Muttenz, Switzerland). The applied sample volumes were in the range of 4 *µ*L. Detection of the solute zones was performed with the use of UV-Vis Scanner (Camag, Muttenz, Switzerland) at 254 nm and 270 nm of the wavelength.

#### 2.4.3. Chromatogram Development

HPTLC developments were performed in horizontal DS chamber for TLC (model DS-II-10 × 10, Chromdes, Lublin, Poland). Chromatograms were developed after 15 min equilibration of the chamber atmosphere and chromatographic plate with the mobile phase vapours. The migration distance of the mobile phase was 45 mm.

The detection of chromatograms and electrochromatograms has been performed with CAMAG TLC Scanner 4 (CAMAG, Muttenz, Switzerland).

The values of migration distance of solutes were the average of triplicate experiments.

#### 2.4.4. Isolation of Phenolic Compounds from Plant Material

The accelerated solvent extraction (ASE) was applied for isolation of investigated compounds from plant material. ASE was performed using a previously elaborated method published by Kozyra and Skalicka-Woźniak [40]. Dried, powdered inflorescences or herbs (1.0 g) of the selected species were subjected to accelerated solvent extraction in ASE 100 apparatus (Dionex, Sunnyvale, CA, USA). The following extraction conditions were used: 70% methanol as extraction solvent, temperature of 85°C, flush volume 60%, purge time: 150 s, number of cycles: 3, and duration of a cycle: 10 min. The obtained extracts were concentrated under reduced pressure at 50°C, dissolved in methanol, and filtered (PTFE Cronus Syringe Filter, 25 mm, 0.45 *μ*m) into 10 mL calibrated vials as follows: (1) *Carduus carlinoides* herb, (2) *Carduus carlinoides* flowers, (4) *Carduus nigrescens* flowers, (6) *Cirsium canum* flowers, and (7) *Cirsumdec causatum* flowers.

The obtained methanol extract from flowers of *Carduus carlinoides* (sample 3) and *Carduus nigrescens* (sample 5) was evaporated to dryness and dissolved in 100 mL hot water, was left in a refrigerator for 24 h, and then filtered through a paper filter. After the separation of ballast extract, the residue was extracted with diethyl ether and then washed with 5% aqueous sodium bicarbonate solution. The ether layer obtained in such a manner was enriched in phenolic compounds.

## 3. Results and Discussion

**Figure 1:**
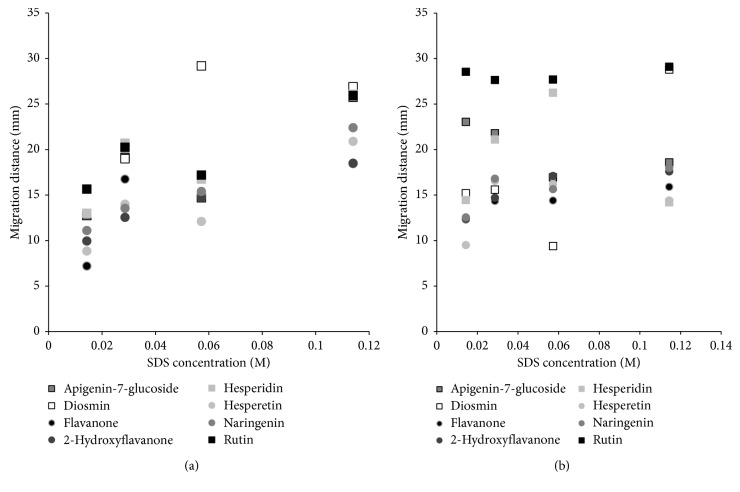
Migration distance of flavonoids vs. SDS concentration in the mobile phase for HPTLC (a) and PPEC (b) experiments. The mobile phase: butanol (0.89 M; 8.14% v/v), 10% acetonitrile, and SDS in the range of 0.0143 to 0.1144 M aqueous buffer of pH 7.0 (0.88 mM of citric acid and 8.23 mM of disodium hydrogen phosphate). The stationary phase: HPTLC RP-18W. TLC: experiment time 20 min. PPEC: polarization voltage 800 V; experiment time 8 min.

**Figure 2:**
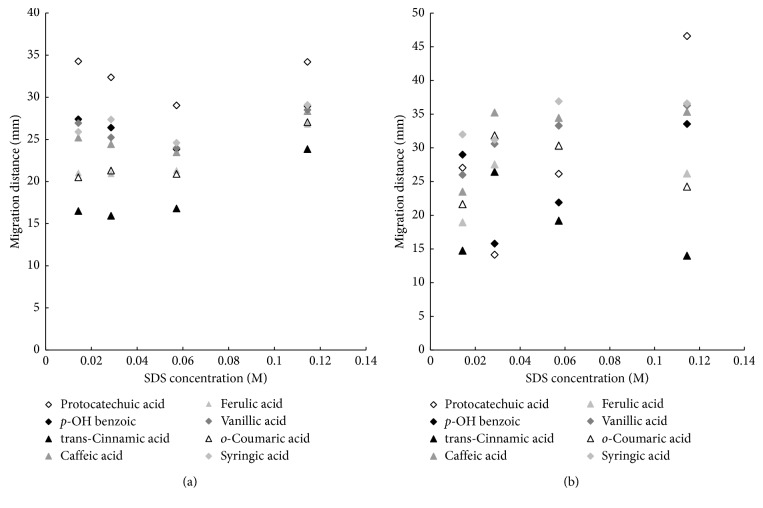
Migration distance of phenolic acids vs. SDS concentration in the mobile phase for HPTLC (a) and PPEC (b) experiments. The mobile phase: butanol (0.89 M; 8.14% v/v), 10% acetonitrile, and SDS in the range of 0.0143 to 0.1144 M aqueous buffer of pH 7.0 (0.88 mM of citric acid and 8.23 mM of disodium hydrogen phosphate). The stationary phase: HPTLC RP-18W. TLC: average experiment time 20 min. PPEC: polarization voltage 800 V; experiment time 8 min.

In the first stage of the experiment, we determine the effect of the concentration of surfactant in the mobile phase on the retention of investigated solutes. The results are presented in Figures 1(a) and 1(b) (flavonoids, (a) HPTLC and (b) PPEC) and Figures 2(a) and 2(b) (phenolic acids, (a) HPTLC and (b) PPEC). Since the tests premise investigation of separation mechanism, the highest content of surfactant (above the CMC value) in the mobile phase has been established according to literature data [41], and it has been progressively reduced. Thus, the concentration of the surfactant in the mobile phase has been in the range from 0.0143 to 0.1144 M. Remaining components are as follows: 8.14% butanol (0.890 M), 10% acetonitrile, 0.1144 M SDS, and buffer of pH 7.0 (0.88 mM of citric acid and 8.23 mM of disodium hydrogen phosphate). Since the solvent front migration distance is difficult to determine in PPEC, therefore, the solute migration distances for both techniques are presented and compared in all paper.

For the planar chromatography mode (Figure 1(a)), the increasing amount of surfactants in the mobile phase results in the formation of micelles in this phase. Their organic parts interact with a nonpolar flavonoid molecule via hydrophobic interactions. However, these types of interactions are also possible between sorbent hydrocarbon chain (C-18) and nonpolar part (C-10) of SDS. As a result, a new kind of stationary phase is formed. It bears the negative charge onto its surface and it has the hydrophilic character [13]. The number of surfactant monomers which modifies the stationary phase is constant. Therefore, the rising total content of SDS in the mobile phase leads to enhancement of micelle concentration in the mobile phase and increases the interaction of solute and micelle and the mobile phase strength. Such behaviour is typical of micellar liquid chromatography [42]. The elongation of flavonoid migration distances with the increase of SDS concentration presented in Figure 1(a) indicates such mode of the retention mechanism. The best separation of flavonoids is obtained when the lowest concentration of SDS (0.0143 M) is applied. The order of substances is as follows: rutin (15.65 mm) > diosmin (15.60 mm) > hesperidin (13.00 mm) > apigenin-7-glucoside (12.75 mm) > naringenin (11.10 mm) > 2-hydroxyflavanone (9.95 mm) > hesperetin (8.85 mm) > flavanone (7.20 mm). The main trend of the migration order is as follows: flavonoid glycosides (hydrophilic compounds of higher molecular mass) migrate longer distances than flavonoids without sugar groups (aglycones, more hydrophobic solutes). So the most hydrophobic compound (flavanone) has the shortest migration distance. This fact is consistent with literature data [27]. Two fronts of the mobile phase are observed during HPTLC experiments. It is in accordance with literature data [43]. Nevertheless, this fact has insignificant effect on the solute retention.

Considering PPEC mode of flavonoid separation (Figure 1(b)), the abovementioned change of the stationary phase character takes place during the prewetting process. Thus, sorbent surface is hydrophilic, bearing negative charge before starting the experiment. Additionally, only one from PPEC effects, namely, electroosmotic effect (electroosmotic flow, EOF), influences the solute migration distances [29]. The second effect (electrophoretic) present in PPEC is insignificant since the flavonoid molecule is neutral. Consequently, regarding mechanism of separation in PPEC, migration distances of flavonoids are the result of electroosmotic flow toward cathode and partition of micelle-compound complexes between the stationary and mobile phases (mentioned earlier at HPTLC part). Since rising SDS concentration in the mobile phase in HPTLC mode results in the elongation of the solute migration distances, the same effect is expected also in PPEC (Figure 1(b)). The SDS concentration in the eluent affecting good separation of solute zones is higher than at HPTLC mode (0.0572 M). The order of the compounds according to the decreasing migration distances for this concentration is as follows: rutin (27.70 mm) > hesperidin (26.25 mm) > diosmin (20.35 mm) > apigenin-7-glucoside (16.95 mm) > 2-hydroxyflavanone (17.10 mm) > hesperetin (16.20 mm) > naringenin (15.65 mm) > flavanone (14.40 mm). This sequence for the majority of solutes is consistent with HPTLC results. Flavonoid glycosides exhibit weaker retention compared to flavonoids without sugar group. This fact is also squared with literature data from HPTLC and CE [25, 27]. Due to the fact that the highest concentration of SDS in the mobile phase resulted in too high current during PPEC process, to perform all experiments at the same conditions the polarization voltage has been reduced to 800 V. This resulted in shortening of migration distances. Nevertheless, the migration distances of flavonoids in PPEC are longer in comparison to those in HPTLC experiments. In addition, the PPEC test time is considerably shorter than HPTLC (8 and 20 min., respectively).

The phenolic acids are the second group of investigated compounds. Contrary to flavonoids, they undergo ionisation process. Therefore, phenolic acid migration distances in HPTLC are the result of both hydrophobic effect between micelles and phenolic acid anion (in the mobile phase) and its interaction with the stationary phase surface. Consequently, the effect of SDS concentration in the eluent on the retention of phenolic acid in the HPTLC technique is more complicated in comparison to presented before flavonoid. The plots of these solute migration distances vs. SDS content in the eluent are shown in Figure 2(a). The increase of the SDS content in the range of 0.0143–0.0572 M enables the penetration of solutes depth into the stationary phase, which results in adsorption and diminution of migration distances [13]. The shortest migration distances of the majority of solutes are observed when 0.0572 M of SDS is present in the mobile phase. Further increasing of SDS content results in the diminution of solute retention by stronger interaction in the mobile phase. Unfortunately, it worsens the phenolic acid separation. The order of the phenolic acid migration distance decreasing for the smallest SDS amount in the eluent is as follows: protocatechuic (34.27 mm) > *p*-hydroxybenzoic (27.40 mm) > vanillic (26.93 mm) > syringic (25.90 mm) > caffeic (25.20 mm) > ferulic (21.03 mm) > *o*-coumaric (20.50 mm) > *trans*-cinnamic (16.50 mm). This order is in accordance with the enhancement of the solute hydrophobicities (see Table 1).

Contrary to HPTLC in PPEC mode (Figure 2(b)), the increase in the concentration of SDS in the mobile phase diminishes the phenolic acid retention for all surfactant concentration range. This is the result of the high electroosmotic flow, on one hand, and electrophoretic effect on the other. The order of the acid retention decreasing for the lowest concentration of SDS is as follows: syringic (32.00 mm) > *p*-hydroxybenzoic (29.00 mm) > protocatechuic (27.05 mm) > vanillic (26.00 mm) > caffeic (23.50 mm) > *o*-coumaric (21.60 mm) > ferulic (18.95 mm) > *trans*-cinnamic (14.75 mm). This order is inconsistent with the presented HPTLC results. However, for the majority of solutes, it complies with enhancement of their hydrophilicity (see Table 1). Comparing the HPTLC and PPEC results, it should be noted that migration distances of the majority phenolic acids are longer in the HPTLC technique (exception for *p*-hydroxybenzoic, syringic, and *o*-coumaric acid). It is the effect of the opposite direction of electrophoretic movement of phenolic acid anion in relation to electroosmotic flow in PPEC. As it has been mentioned earlier, the content of SDS (0.1144 M) in the mobile exceeded 50 mA (the highest allowed electric current) and due to this polarization voltage used was only 800 V. It limits the possibilities of the PPEC system. However, the time of PPEC experiment is shorter than HPTLC, and separation of solute zones is better for the previous technique.

**Figure 3:**
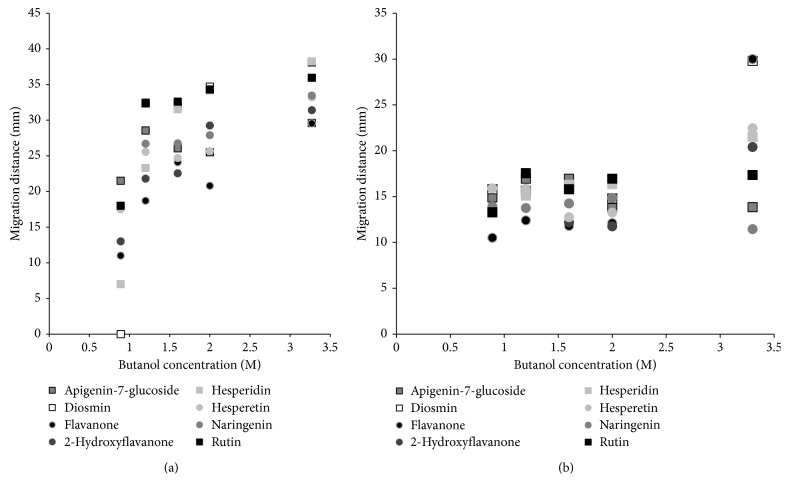
Migration distance of flavonoids vs. butanol concentration in the mobile phase for HPTLC (a) and PPEC (b) experiments. The mobile phase: butanol (0.89–3.27 M; 8.14–30.00% v/v), 10% acetonitrile, 0.1144 M SDS, and aqueous buffer of pH 7.0 (0.88 mM of citric acid and 8.23 mM of disodium hydrogen phosphate). The stationary phase: HPTLC RP-18W. TLC: experiment time 20 min. PPEC: polarization voltage 800 V; experiment time 8 min.

**Figure 4:**
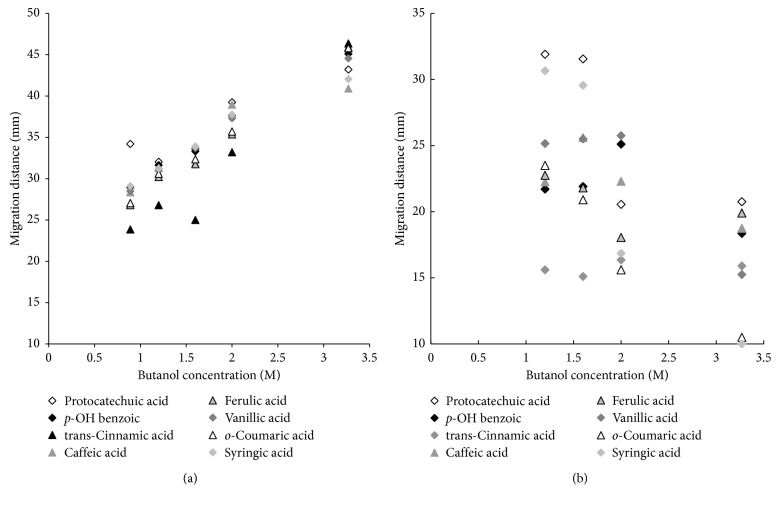
Migration distance of phenolic acids vs. butanol concentration in the mobile phase for HPTLC (a) and PPEC (b) experiments. The mobile phase: butanol (0.89–3.27 M; 8.14–30.00% v/v), 10% acetonitrile, 0.1144 M SDS, and aqueous buffer of pH 7.0 (0.88 mM of citric acid and 8.23 mM of disodium hydrogen phosphate). The stationary phase: HPTLC RP-18W. TLC experiment time: 20 min. PPEC: polarization voltage 800 V; experiment time 8 min.

Since the results achieved for both classes of compounds were inadequate, in the next stage of experiment the effect of organic solvent changes in the mobile phase on solute migration distances has been investigated. The results are presented in Figures 3(a) and 4(a) (flavonoids and phenolic acids, HPTLC) and Figures 3(b) and 4(b) (flavonoids and phenolic acids, PPEC). The range of organic alcohol in the eluent is 0.89–3.27 M (8.14–30.00% v/v). Remaining components are 10% acetonitrile, 0.1144 M SDS, and buffer of pH 7.0 (0.88 mM of citric acid and 8.23 mM of disodium hydrogen phosphate). The presence of surfactant (SDS) promotes the solubility of butanol in water (solubilisation effect), which enables the preparation of a solution containing 30% v/v butanol (without surfactant, the maximum concentration of butanol in water is approximately 8%).

In the HPTLC system (Figure 3(a)), the retention of flavonoids diminishes when the mobile phase becomes less polar (at higher butanol content). This is the result of decrease of the eluent polarity and simultaneously enhancement of its hydrophobicity and alkalinity. The factors described above increase the affinity of the substances to the mobile phase; thus, their migration distances are elongated. Additionally, the butanol structure is similar to surfactant (polar and non-polar ends); thus, it is incorporated to the micelles forming mixed entities [13, 15]. In addition, the presence of acetonitrile in the mobile phase, according to the literature data, decreases the content of SDS necessary to reach CMC [44].

Such behaviour promotes the interaction of flavonoid and micelle in the eluent and reduces solute retention.

The best separation of the solute zones is observed for the mobile phase containing 1.2 M (10.98% v/v) of butanol. The sequence of bands, by decreasing the distance of migration, is as follows: rutin and diosmin (32.35 mm) > apigenin-7-glucoside (28.55 mm) > naringenin (26.7 mm) > hesperetin (25.6 mm) > hesperidin (23.3 mm) > 2-hydroxyflavanone (21.8 mm) > flavanone (18.7 mm). It differs from the above-described concentration of butanol (30% v/v) and presented earlier data (solute migration distance vs. SDS concentration dependence).

The migration distance of the solutes in the PPEC technique (Figure 3(b)) slightly changes when the concentration of butanol in the mobile phase increases from 0.890 M to 2.00 M, thereafter subsequently rises when the concentration reaches 3.27 M. The electroosmotic flow, electrophoretic effect, and solute partition between mobile and stationary phases are responsible for the solute movement in PPEC according to literature data [28–39]. Since the flavonoids are not ionised, as it has been mentioned before, the electrophoretic effect slightly affects these solute migration distances. Thus, the flavonoid migration distances in the PPEC system are the result of only electroosmotic flow and partition effect. Subsequently, since both butanol and micelles are viscous and according to Smoluchowski equation viscosity is inversely proportional to the electroosmotic flow; thus, the increase of the content of butanol in the mobile phase deteriorates EOF. Due to this, the migration distances of flavonoids are shorter in comparison with the HPTLC technique.

Significant elongation of the solute migration distances is observed for the highest concentration of butanol in the mobile phase. It is the result of strong hydrophobic interaction between solute and mixed micelles in the mobile phase. For this content of butanol in the eluent, the order of reduction in the migration distance of the flavonoids is as follows: flavanone (30 mm) and diosmin (29.8 mm) > hesperetin (22.45 mm) >  hesperidin (21.5 mm) > 2-hydroxyflavanone (20.4 mm) > rutin (17.35 mm) > apigenin-7-glucoside (13.85 mm) > naringenin (11.45 mm). This sequence differs from HPTLC one. Longer migration distance of flavanone compared to 2-hydroxyflavanone is the result of the highest hydrophobicity of this compound (see log *P* values in Table 1).

Interestingly, the content of butanol in the mobile phase also affects the selectivity of the system. Thus, for the smallest concentration of butanol (0.89 M, 8.14% v/v) in the mobile phase, the order of flavonoids migration distances differs from the presented before. It started from the weakest retention as follows: hesperetin (15.9 mm) > diosmin (15.8 mm) > apigenin-7-glucoside (14.9 mm) > naringenin (13.9 mm) > hesperidin (13.2 mm) > 2-hydroxyflavanone(13.4 mm) > rutin (13.3 mm) > flavanone (10.5 mm). In this case, longer migration distances are observed for a majority of flavonoid glycosides (diosmin and apigenin-7-glucoside) in comparison with flavonoids without sugar groups (naringenin, 2-hydroxyflavanone, and flavanone). Similarly to previous butanol concentration, the order of the pair hesperetin (aglycone) and hesperidin (flavonoid glycoside) is opposite to presented earlier statement. Thus, aglycone migrates longer distance than flavonoid glycoside. As previously described, the PPEC experiment time is shorter in comparison to HPTLC one.

The effect of butanol content in the mobile phase on the retention of phenolic acids in HPTLC system is presented in Figure 4(a). The migration distances of phenolic acids enhance when butanol content in the mobile phase increases. It is consistent with results presented before for flavonoids and HPTLC mode. The order of the solute elution for 3.27 M butanol in the eluent is as follows (started from the longest migration distances): *trans*-cinnamic acid (46.35 mm) > ferulic acid + *o*-coumaric acid (45.90 mm) > *p*-hydroxybenzoic acid (45.10 mm) > vanillic acid (44.55 mm) > protocatechuic acid (43.20 mm) > syringic acid (42.05 mm) > caffeic acid (40.90 mm). It is compliant with the diminution of solute hydrophobicity (Table 1). Thereby, the most hydrophobic is *trans*-cinnamic acid bearing unsaturated alkyl chain attached to the aryl ring without additional polar (hydroxyl) groups in its molecule. Presence of polar group or groups in the aryl ring enhances the polarity of solute and reduces migration distance. Therefore, the order of migration distance decreasing for the derivatives of cinnamic acid is succeeding: cinnamic (46.35 mm) > ferulic (45.9 mm) > *o*-coumaric (45.8 mm) > caffeic (40.9 mm). The latter acid possesses two hydroxyl groups in the aryl ring and consequently it is the most polar one. Considering the chemical structure of benzoic acid derivatives, the presence of various groups attached to the aryl ring also affects the migration distances but in different ways comparing to cinnamic acid derivatives. The *p*-hydroxybenzoic acid (migration distance 45 mm) bearing only one polar group in the aryl ring is less hydrophilic than vanillic acid (two groups of various polarities in benzene ring; migration distance 44.55 mm). The latter one is in turn more hydrophobic in comparison to protocatechuic acid (two polar groups in the aromatic ring; migration distance 43.2 mm). Consequently, it is less hydrophilic than syringic acid (aryl group with three groups of various polarities, migration distance 42.05 mm).

The migration distance of phenolic acids vs. butanol concentration in the eluent for PPEC system is presented in Figure 4(b). Since phenolic acids are ionisable; thus, in PPEC systems, they undergo electrophoretic effect besides the partition between the mobile and stationary phases. At the experiment conditions (the mobile phase buffer pH equal to 7.0), phenolic acids form anions and their electrophoretic movement is against the electroosmotic flow (EOF, the mobile phase movement). In addition, as it has been mentioned before, the increase in butanol concentration in the mobile phase causes the enhancement of its viscosity, decreases the dielectric constant of eluent [15], and reduces EOF. All these effects result in diminution of phenolic acid migration distances.

For the highest concentration of butanol in the eluent (3.27 M), the order of reduction of the phenolic acid migration distances is as follows: protocatechuic acid (20.75 mm) > ferulic acid (19.90 mm) > caffeic acid (18.75 mm) > *p*-OH benzoic acid (18.35 mm) > *trans*-cinnamic acid (15.90 mm) > vanillic acid (15.25 mm) > *o*-coumaric acid (10.50 mm) > syringic acid (10.00 mm). This order is in accordance with the increase in the log *P* values and p*K*_A2_ (see Table 1) for the majority of acids (the exceptions are *trans*-cinnamic and *o*-coumaric acids, which possess only one ionisable group).

**Figure 5:**
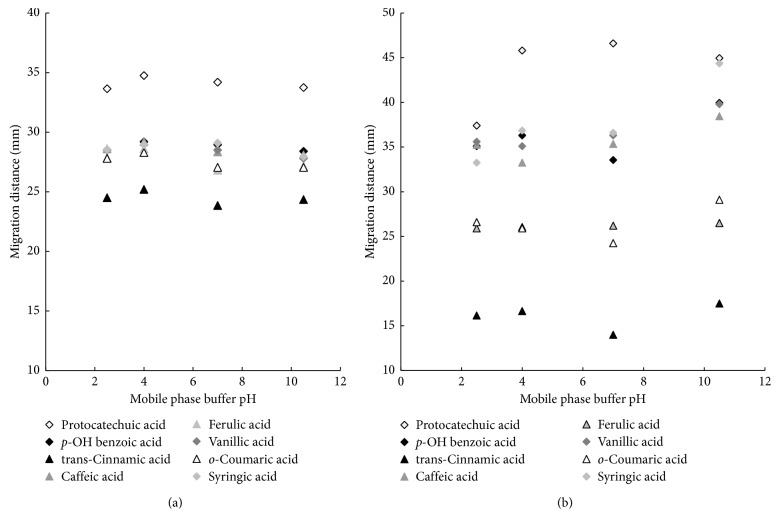
Migration distance of phenolic acids vs. mobile phase buffer pH in the mobile phase for HPTLC (a) and PPEC (b) experiments. The mobile phase: butanol (0.89 M; 8.14% v/v), 10% acetonitrile, 0.1144 mM SDS, and aqueous buffer (citric acid, tris(hydroxymethyl)aminomethane, glycine 0.8 mM titrated to proper pH with KOH or HCl). The stationary phase: HPTLC RP-18W. TLC: experiment time 20 min. PPEC: polarization voltage 800 V; experiment time 8 min.

As it has been mentioned before, contrary to flavonoids, the phenolic acids are ionisable compounds; thus, in the next stage of experiments, the relationship of their migration distances vs. the mobile phase buffer pH for both techniques is investigated. The influence of the mobile phase buffer pH on retention of investigated solutes is presented in Figures 5(a) and 5(b) for HPTLC and PPEC techniques, respectively. The used range of buffer pH is 2.5–10.5.

The mobile phase buffer pH insignificantly affects the retention of phenolic acids in the HPTLC technique (Figure 5(a)). The weakest retention of investigated solutes (the longest migration distances) is observed at buffer pH equal to 4.0. This value is close to p*K*_A_ of tested acids (see Table 1); hence, hydrophobicity changes of investigated solutes are responsible for such behaviour. The order of the retention enhancement for the system with the buffer of pH 10.5 is as follows: protocatechuic acid (33.75 mm) > *p*-hydroxybenzoic acid (28.40 mm) > caffeic acid (28.10 mm) > syringic acid (27.95 mm) > vanillic acid (27.80 mm) > ferulic acid + *o*-coumaric acid (27.15 mm) > *trans*-cinnamic acid (24.15 mm). This order for the majority of phenolic acids is in accordance with the increasing of log *P* values (see Table 1).

Regarding the PPEC system, the increase in the mobile phase buffer pH enhances the solute migration distances (Figure 5(b)). The longest migration distances of the majority of solutes are noticed for the eluent buffer pH equal to 10.5. As it was reported earlier for the PPEC technique, the mobile phase buffer pH strongly affects the electroosmotic flow, and the highest movement of the eluent is observed when high pH value is applied [28]. The order of the solute migration for eluent with buffer pH 10.5 is as follows (started from the longest migration distance): protocatechuic acid (44.95 mm) > syringic acid (44.35 mm) > *p*-hydroxybenzoic acid + vanillic acid (39.95 mm) > caffeic acid (38.45 mm) > *o*-coumaric acid (29.10 mm) > ferulic acid (26.50 mm) > *trans*-cinnamic acid (17.50 mm). The chemical structure of investigated solutes affects the order. Thus, derivatives of the benzoic acids (more polar solutes of lower log *P* values) migrate longer distances in comparison with the cinnamic acid derivatives (less polar solutes of higher log *P* values). The sequence of the phenolic acids in PPEC differs from this of HPTLC experiments. In addition, the separation time in PPEC is again shorter than in HPTLC.

**Figure 6:**
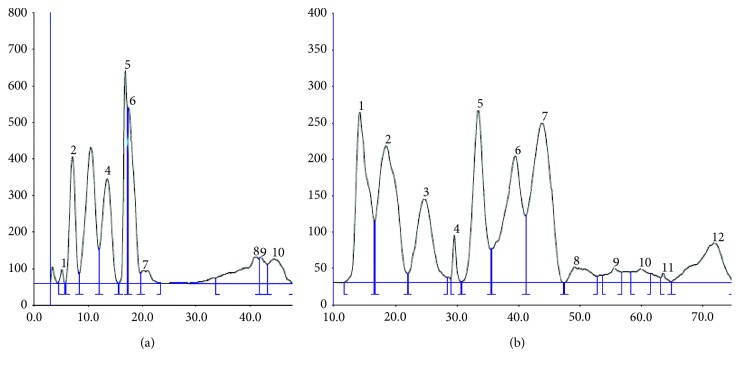
Separation of the mixture of flavonoids and phenolic acids with the use of HPTLC (a) and PPEC (b) techniques. Experiment conditions: the mobile phase: butanol 1.2 M, acetonitrile 10% v/v, aqueous buffer of pH 7.0 (0.88 mM of citric acid and 8.23 mM of disodium hydrogen phosphate), and 0.040 M of SDS; the stationary phase: HPTLC RP-18W. PPEC: polarization voltage 1400 V; experiment time 8 min; experiment temperature 38^o^C. The order of solutes for HPTLC: (1) rutin, (2) hesperidin; (3) *trans*-cinnamic acid and hesperidin; (4) caffeic acid and *o*-coumaric acid; (5) flavanone; (6) vanillic acid. The order of compounds in the PPEC experiment: (1) flavanone; (2) *trans*-cinnamic acid and hesperetin; (3) *o*-coumaric acid; (4) rutin; (5) hesperidin; (6) vanillic acid; (7) caffeic acid.

The experiments presented above allowed compiling the experimental conditions which lead to the best distinguishing of the mixture of flavonoids and phenolic acids. They allow applying higher polarization voltage for PPEC experiments (1400 V). Comparison of the separation of mixture of 4 flavonoids and 4 phenolic acids with the use of HPTLC and PPEC techniques is presented in Figures 6(a) and 6(b). There are only 6 zones of solutes in chromatogram shown in Figure 6(a) contrary to that in Figure 6(b) in which baseline separation of 7 compounds is presented. In addition, the experiment time in PPEC lasts only 8 min in comparison to 20 min of HPTLC.

**Figure 7:**
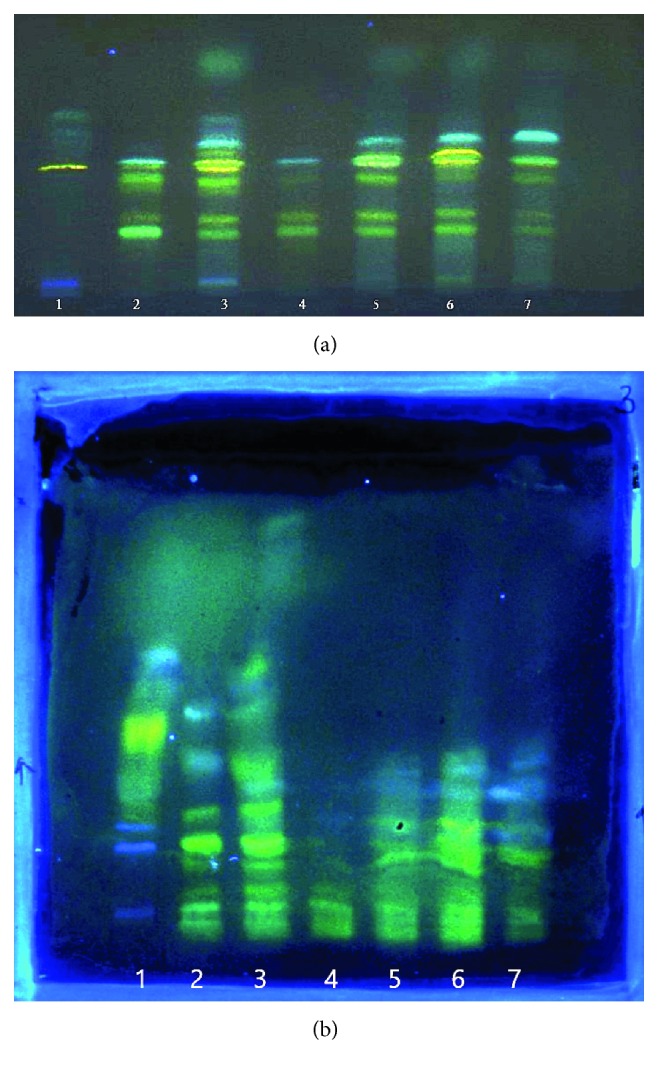
Separation of plant extracts with HPTLC (a) and PPEC (b) techniques. Plant extracts No.: (1) *Carduus carlinoides* herb, (2) *Carduus carlinoides* flowers, (3) *Carduus carlinoides inflorescentia* methanolic extract fraction Fb, (4) *Carduus nigrescens* flowers, (5) *Carduus nigrescens* flowers methanolic extract fraction Fb, (6) *Cirsium canum* flowers, (7) *Cirsum deccusatum* flowers. Experiment conditions: the mobile phase: 0.040 M SDS, pH 7.0 (0.88 mM of citric acid and 8.23 mM of disodium hydrogen phosphate), 1.2 M butanol, and 10% v/v acetonitrile; the stationary phase: HPTLC RP-18W. PPEC experiment: polarization voltage 1400 V; experiment time 8 min. Detection after derivatization at *λ* = 366 nm.

The achieved results encourage us to separate the genuine mixture with the use of system with surfactant and both techniques. Figures 7(a) and 7(b) present the separation of plant extracts performed with PPEC and HPTLC, respectively. Comparing both photos (HPTLC and PPEC separation), the changes in the migration zone orders is observed. More extract solute zones are presented in Figure 7(b) (PPEC experiment). However, the complicity of plant mixture and imperfection of the PPEC technique causes the separation process with HPTLC more efficient. This points to the need to further develop and improve this electromigrational technique. Nevertheless, the PPEC experiment time is considerably shorter (8 min).

A repeatability of experiments performed with the HPTLC and PPEC techniques was compared by statistic evaluation of chosen flavonoid and phenolic acid migration distances. The complete data are presented in Table 2. The mean migration distances of flavanone and rutin investigated in PPEC are shorter in comparison with HPTLC due to its nonionic character. The same parameter for *o*-coumaric and vanillic acids (ionisable compounds) is significantly longer in PPEC. Considering relative standard deviation, RSD, its values are higher for the abovementioned technique. Such a behaviour characterises all electromigrational techniques.

## 4. Conclusions

The results presented above confirm that application of surfactant as the mobile phase additives in the PPEC technique is possible. The equilibration of the chromatographic plate with the mobile phase before the separation process in this mode leads to creation of experimental conditions analogous to those of column techniques. Such a procedure is not applicable in the HPTLC system. Various orders of solute migration in both systems are perceived. The main separation mechanism in PPEC and HPTLC modes is the interaction between system consisting of surfactant (micelles) and separated compound with the stationary and mobile phases. The features such as concentration of SDS, butanol, and pH affect the formation of micelles and separation of investigated solutes in both techniques. Considering PPEC, the high content of SDS results in high current, which limits the polarization voltage applied. Nevertheless, experiment time in this technique is considerably shorter than that in HPTLC. The mixture of solute various types (ionic and nonionic) is satisfactory separated with the use of both techniques. The kind of compound significantly affects the solute distinguishing by PPEC.

Bearing in mind less complicated mixtures, the results presented here support the PPEC advantages: short experiment time, various solutes order, and higher efficiency in comparison with the HPTLC technique. Nevertheless, the application of PPEC for separation of genuine plant extract is still the matter of future.

## Figures and Tables

**Table 1:** Structural formulas and physicochemical properties of investigated compounds.

Flavonoids		
Name	Lipophilicity	Formula
Apigenin-7-glucoside	Log *P* = −0.39 [45]	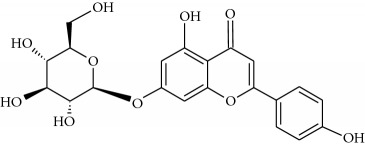
	*K*_w/c_ = 10.13 [46]	

Diosmin	Log *P* = 2.05 [45]	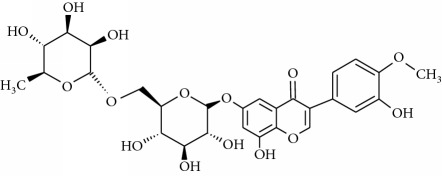

Flavanone	Log *P* = 3.62 [45]	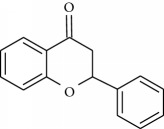
	*K*_w/c_ = 0.16 [46]	

2-Hydroxyflavanone	Log *P* = 3.10 [45]	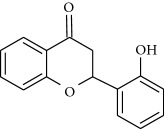

Hesperidin	Log *P* = 1.78 [45]	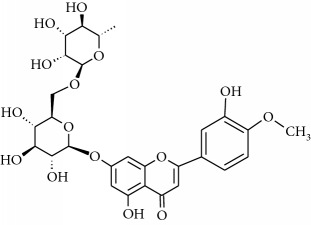

Hesperetin	Log *P* = 2.90 [45]	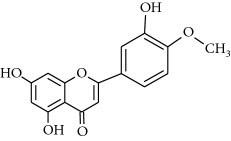
	*K*_w/c_ = 0.02 [46]	

Naringenin	Log *P* = 3.19 [46]	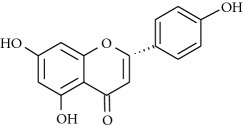
	*K*_w/c_ = 0.55 [46]	
Rutin	Log *P* = 1.76 [45]	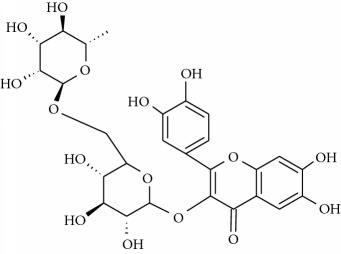
	*K*_w/c_ = 113.47 [46]	

Phenolic acids				
Acid name	p*K*_A1_	p*K*_A2_	Log *P*^*∗*^	Formula
*p*-Hydroxybenzoic	4.25 [47]	9.1	1.42	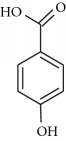

Protocatechuic	4.54 [47]	8.53	1.16	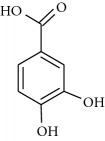

Vanillic	4.04 [47]	9.08	1.33	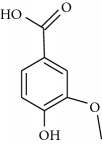

Syringic	4.46 [48]	9.33 [48]	1.13	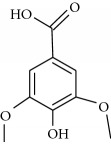

*trans*-Cinnamic	5.5 [45]	—	2.41	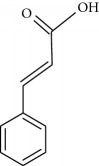

*o*-Coumaric	4.52 [49]	—	1.88	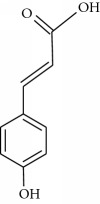
Caffeic	4.48 [48]	8.83	1.42	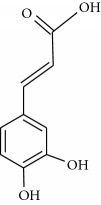

Ferulic	4.38 [48]	8.75	1.64	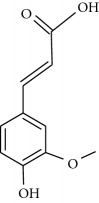

**Table 2:** Statistic evaluation of chosen flavonoid and phenolic acid migration distances in PPEC and TLC techniques.

Solute	*n*	Mean migration distance (mm)	Standard deviation from migration distance	Variance	Confidence interval	RSD%
PPEC						
Flavanone	7	15.01	0.49	0.28	0.4	3.3
Rutin	7	28.88	1.57	2.87	1.0	5.4
*o*-Coumaric acid	7	23.11	1.30	1.97	1.0	4.6
Vanillic acid	7	30.39	1.43	2.38	1.0	4.5
HPTLC						
Flavanone	7	27.20	0.38	0.17	0.1	1.4
Rutin	7	35,89	1.14	1.52	0.3	3.2
*o*-Coumaric acid	7	19.70	0.39	0.18	0.1	2.0
Vanillic acid	7	19.10	0.54	0.34	0.1	3.0

## Data Availability

The data used to support the findings of this study are included within the article.
